# High familial risks in some rare cancers may pinpoint to hidden germline genetics: focus on esophageal, stomach, small intestinal, testis, thyroid and bone cancers

**DOI:** 10.1186/s13053-024-00303-6

**Published:** 2025-02-27

**Authors:** Kari Hemminki, Otto Hemminki, Anni Koskinen, Akseli Hemminki, Asta Försti

**Affiliations:** 1 Biomedical Center, Faculty of Medicine, Charles University, Pilsen, 30605 Czech Republic; 2 Division of Cancer Epidemiology, German Cancer Research Center (DKFZ), Im Neuenheimer Feld 580, D-69120 Heidelberg, Germany; 3 Department of Urology, Helsinki University Hospital, Helsinki, Finland; 4 Cancer Gene Therapy Group, Translational Immunology Research Program, University of Helsinki, Helsinki, Finland; 5 Department of Otorhinolaryngology- Head and Neck Surgery, Helsinki University Hospital and University of Helsinki, Helsinki, Finland; 6 Skin and Allergy Hospital, Helsinki University Hospital and University of Helsinki, Helsinki, Finland; 7 Comprehensive Cancer Center, Helsinki University Hospital, Helsinki, Finland; 8 Hopp Children’s Cancer Center (KiTZ), Heidelberg, Germany; 9 Division of Pediatric Neurooncology, German Cancer Research Center (DKFZ), German Cancer Consortium (DKTK), Heidelberg, Germany

**Keywords:** Familial risk, Germline genetics, Constitutional variants, Heredity

## Abstract

**Background:**

Germline genetic susceptibilities of rare cancers of the esophagus, stomach, small intestine, testis, (nonmedullary) thyroid gland and bone with high familial risks are not well known. Here, we use familial risk data from the Swedish Family-Cancer Database which contains records of cancers in Swedish families obtained over a century. We compare familial risks for offspring diagnosed with any of these cancers when their parent had or had not that cancer. We review the global literature of the reported constitutional variants that may explain part of the familial risk.

**Main body:**

Familial risks for esophageal and stomach cancers are about 2.0 and apart from early-onset stomach cancer few high-risk variants are known. Genetic studies may be hampered by dominant environmental risk factors for these cancers. Small intestinal carcinoids have a very high familial risk (28 between siblings) but no high-risk genes have been identified to explain this. Low-risk polygenic variants have been identified. Small intestinal adenocarcinoma is a manifestation in Lynch syndrome. Testicular and thyroid cancers are characterized by high familial risk (about 5) which may be explained largely by a polygenic background, although thyroid cancer is a component in a number of rare cancer syndromes. Several predisposing genes have been identified for bone cancer (familial risk 7).

**Conclusions:**

The discussed cancers are rare and they present with a relatively high familial risk, in spite of lacking identified high-penetrant constitutional variants. It is possible that the polygenic component, already recognized for testis cancer, is stronger than previously expected. Thus polygenic models with rare high/moderate- and low-risk variants could fit the familial risk and shape the germline genetic landscape of these cancers. Polygenic background may have clinical implications.

## Introduction

Female breast cancer accounted for over 25% of all female cancers in Europe and North America in 2022 according to the Cancer Today database of the International Agency for Cancer (IARC) (https://gco.iarc.who.int/today/en/dataviz/tables?mode=population&sexes=1). According to the same source prostate cancer accounted for barely under 20% of all male cancers. If we additionally include colorectal cancer as a third common cancer, the large share of these cancers probably explains why much of the literature on germline genetics of cancer has focused on these cancers. For breast and colorectal cancers the discoveries of BRCA1/2 and mismatch repair genes with major clinical impact stimulated a further search of novel constitutional variants [1, 2]. These genes were found in families of many affected individuals although the overall population-level familial risk in these cancers is not particularly high (Table 1) [3].

**Table 1:** Concordant familial risks when one or at least two probands were diagnosed with cancer

	One family member diagnosed with concordant cancer						Two or more family members diagnosed with concordant cancer									
Cancer site	O1	SIR	95% CI		O2		%^1)^	SIR		95% CI						
Esophagus	73	**2.59**	**2.03**	**3.25**	0											
Stomach	401	**1.83**	**1.65**	**2.01**	10		2.4	**5.6**		**2.64**		**10.25**				
Small intestine	52	**5.64**	**4.21**	**7.40**	2		3.7	**112.3**		**10.6**		**413.2**				
Testis	158	**5.24**	**4.45**	**6.12**	0											
Thyroid gland, nonmedullary	136	**4.33**	**3.63**	**5.12**	0											
Bone	9	**6.93**	**3.14**	**13.2**	0											
Colorectum	7233	**1.70**	**1.66**	**1.74**	417		5.4	**2.8**		**2.5**		**3.0**				
Breast	15,805	**1.74**	**1.71**	**1.76**	913		5.5	**2.5**		**2.3**		**2.7**				
Prostate	21,688	**2.20**	**2.17**	**2.23**	2550		11.7	**3.7**		**3.6**		**3.9**				

O1 = Observed, 1 affected proband; O2 = Observed, 2 or more affected probands; SIR = Standardized incidence ratio; CI = Confidence intervals.^1)^ % O2 of O1 + O2

In this review we focus on rare cancers of the esophagus, stomach, small intestine, testis, (nonmedullary) thyroid gland and bone with a relatively high familial risk which should enhance the likelihood of finding underlying constitutional variants. However, as cancer is a multifactorial disease, in the selection of a study population for germline variants one has to consider also non-genetic causes. For example, lung cancer has an equal familial risk as the common cancers of breast, prostate and colorectum, but it is thought to be mainly caused by cigarette smoking; the confirmed germline contribution is small and includes genes, such as the nicotinic acetylcholine receptor (*CHRNA3*) gene, which influences smoking dependence and levels [4, 5]. Similarly, for gastric and cervical cancers microbial infections are thought to be the main causes of disease [6]. Finding constitutional genetic variants or their interactions with the dominant environmental or infections causes may be challenging. As for familial risks, we rely on data from the Swedish Family-Cancer Database [3]. In addition to the familial risk, also case numbers define the likelihood of being able to collect sample sets with reasonable statistical power for gene finding efforts. For genetic studies we searched the global literature for likely explanations. For credible findings regarding constitutional variants, evidence should be shown that they are more frequent in cases compared to controls, using either concurrent controls or referring to available databases, such as ClinVar [7] or gnomAD (https://gnomad.broadinstitute.org). At the end we discuss the results also in terms of genetic models which have recently been expanded to combined risk estimates between monogenic and polygenic models [8, 9].

## Familial risks

The Swedish Family-Cancer Database is a reliable source of familial risks as from 1932 onwards newborns have been registered with their parents, totaling some 16 million individuals [10]. Cancers for the complete national population are derived from the Swedish cancer registry with practically complete coverage [11].

Familial cancer can be defined through the occurrence of the same cancer in two or more family members. Here we consider only first-degree relatives, parents and offspring at age 20 to 84 years. Familial relative risk (shortly ‘familial risk’) is presented as standardized incidence ratio (SIR) which has been adjusted for a number of possible confounding factors [3]. Risks are calculated for the offspring case when parents are probands; siblings are used also as probands if a parent is also affected in ‘multiplex’ families. For case numbers, only offspring/sibling cases were counted, thus including probands (parents) would double the number of familial cases.

In Table 1 we have collected some rarer cancers for which familial risks are relatively high [3]. As reference familial risks of colorectal, breast and prostate cancers are included; these have large numbers of families with two or more probands (multiplex families) which are almost lacking in the rarer cancers.

### Constitutional variants

Pathogenic variants in ‘classical’ cancer susceptibility genes are rare in the population (allele frequencies < 1/1000) and the risk in carriers of the variants is high (> 5-fold) [12]. After the year 2000, ‘the common disease-common variant’ paradigm was adopted in non-communicable disease genetics which was applied to genome-wide association studies. In cancer, hundreds of formally significant loci have been found, which typically have a high allele frequency (> 10%), low risk (< 1.5) and unknown function [12].

The incidence of esophageal cancer has modestly increased in Sweden during the past 50 years, but with opposite trends for increasing adenocarcinoma and decreasing squamous cell carcinoma incidence [13]. The risk factors for these are different, obesity and gastro-esophageal reflux disease for the former and smoking and alcohol for the latter [14]. Familial cases are rare but the risk is relatively high (2.6, Table 1). However, as no multiplex families are found among the Swedish families the chances of detecting novel constitutional variants is not large; a partial contributor may be the multifactorial causation of this cancer. Studies in germline genetics have covered small numbers of patients showing some 10% pathogenic variant frequency, including the DNA damage response genes *BRCA1, BRCA2, PALB2 *and *ATM* [15]. In esophageal adenocarcinoma the frequency of variants is equal and also *CHEK2* variants were reported [16].

In stomach cancer a somewhat higher frequency (up to 15%) of constitutional pathogenic variants have been reported but the genes resemble those found in esophageal cancer [15]. The early onset hereditary diffuse form is linked to germline mutations in two genes which are a part of the epithelial adherens junction complex, CDH1 (cadherin 1) and CTNNA1 (catenin alpha 1) [17]. Familial risk in stomach cancer is modest (Table 1) and as H. pylori infections are a major cause of this disease, thus the prospects of finding novel constitutional high-risk variants appear meager [18].

Small intestinal cancer constitutes two main histological types: carcinoid (neuroendocrine) tumor and adenocarcinoma, the incidence proportions of which in Sweden have been about 2:1 and increasing recently to 3:1 [19]. According to Table 1 familial risks are among the highest, 5.6, but the case numbers in the whole country were only 52. In a family study among a total of the 1799 small intestinal cancer (SIC) cases, 1.1% had a sibling with SIC; SIR was 11.8 [20]. The SIR of concordant carcinoid histology among siblings was 28.4 and in parent-child pairs it was 9.9. According to this study, familial adenocarcinomas were rare but these are a known manifestation of Lynch syndrome [21]. It is possible that the high risk in multiplex families is related to this syndrome.

Linkage analysis and whole-exome sequencing in SIC families identified a germline 4-bp deletion in the gene inositol polyphosphate multikinase (*IPMK*), which truncates the protein [22]. This mutation was detected in all 11 individuals with SIC and in 17 of 35 family members of an unknown status. A small European family study found no such mutations nor chromosomal deletions at the IPMK locus [23]. A Swedish study of 24 individuals from 15 families identified monoallelic germline mutations in the *MUTYH* gene but found no mutations in *IPMK* [24]. The *MUTYH* variant was found in individual patients from two different families but not in all affected individuals of these families. While the MUTYH mutation may be the causal variant it appears that much of the high familial risk remains unexplained.

Familial risk of testicular cancer (testicular germ cell tumor) is 5.2 with a familial proportion of 1.9%; no multiplex families were identified (Table 1). A genome-wide association study of 730 cases and 1400 controls found several significant loci defined by single nucleotide polymorphisms (SNPs) [25]. The most promising association was on chromosome 12 mapping to the *KITLG* gene, which encodes the ligand for the membrane-bound receptor tyrosine kinase KIT [25]. The three best susceptibility loci together account for 10% of the familial risk for testicular cancer. The Testicular Cancer Consortium assembled 10,156 and 179,683 men with and without testicular cancer [26]. This large genome-wide study identified 22 novel susceptibility loci, bringing the total to 78. Combined these would account for 44% of the familial risk [26]. The authors used the STRING protein–protein interaction network and could outline three interconnecting pathways promoting testicular cancer: male germ cell development within its somatic niche, regulation of chromosomal division and structure, and mRNA translation [26]. Another polygenic risk study estimated that a score of 196 SNPs would account for 54% the familial risk [27].

Later strong evidence for the polygenic etiology of familial testicular cancer was presented from the UK [28]. A Polygenic Risk Score (PRS) was constructed using 37 susceptibility SNPs which compared 236 familial and 3931 sporadic cases, and large number of controls. The susceptibility SNPs were significantly enriched in familial compared to sporadic cases (*p* = 0.0001). Importantly, a large majority of familial cases (84–100%) could be attributed to polygenic enrichment [28].

Curiously, for testicular cancer, one of the most familial cancer, only the polygenic model is firmly supported by the literature, and the above paper demonstrates that the polygenic component accounts for an overwhelming share of the familial clustering [28].

Non-medullary thyroid cancer (here thyroid cancer) has a high familial risk of 4.3 and familial proportion of 2.3%; no multiplex families were identified (Table 1). A joint study from the five Nordic countries addressed histology-specific familial risks in non-medullary thyroid cancer and reported relative risks for concordant papillary and follicular forms at 3–5, 28 when two other family members had papillary cancer and 23 for papillary cancer in twins [29]. Not enough concordant cases were found for the rare anaplastic type but in family pairs of papillary-anaplastic cancers the risk was 5.

Thyroid cancer is a component in more than a dozen cancer syndromes, such as familial adenomatous polyposis and Cowden syndrome [30]. The syndromes are probably extremely rare as no multiplex thyroid cancer families were found in our family studies. At least 12 genes have been associated with these syndromes, including *APC, DICER1, FOXE1, HABP2, NKX2–1, PRKAR1A, PTEN, SDHB, SDHD, SRGAP1, CHEK2*, and Sect. 23 [30]. In non-syndromic thyroid cancer several susceptibility genes have been identified or proposed: *FOXE1, HABP2, NRG1, SRGAP1, DIRC3, TITF1/ NKX2.1 *and *PTCSC3* [30]. Anaplastic thyroid cancer is a rare but very aggressive cancer [31]. Germline genetics in anaplastic cancer are very rare but it may be a manifestation of Dicer 1 syndrome with a constitutional *DICER1* mutation [32].

Bone tumors are rare and overall show two age maxima, one before age 20 (common for osteosarcoma and Ewing sarcoma) and the other at around 75 years (chondrosarcoma and osteosarcoma) [33]. Risk factors are not well known but ionizing radiation, immunosuppression and genetic causes (Li-Fraumeni syndrome) are some. In a large analysis of constitutional variants among children and young adults osteosarcoma patients were found to harbor mutations in *CHEK2, TP53, BRCA1 *and *RECQL* [34]. *CHEK2* variants were also common in Ewing sarcoma, in addition to *BRCA1 *and *POLE *variants [34].

## Conclusions

Among the cancers discussed above, familial risk was about 2.0 for esophageal and stomach cancers and for these the identified constitutional variants probably explain little of the familial clustering. Major environmental risk factors are known for both cancers which may mask finding and assigning novel germline variants. Also, the changing incidence trends of the subtypes of these cancers may disfavour genetic studies. Finding novel genetic risk factors and therapeutic targets may be rewarding but as these cancers are estimated to be somewhere between 50% and 75% environmentally caused the short-term emphasis could be in treating H. pylori infections and Barrett esophagus [18, 35].

For the remaining cancers familial risks were high, 4.3–6.9, which should stimulate the search for genetic correlates. However, these are rare cancers and each are presenting with two or more histological types, which may also imply genetic heterogeneity, known to account for at least small intestinal cancers. For testicular and probably for small intestinal carcinoids and thyroid cancer polygenic component is probably an important contributor to the high familial risk [28].

Combined polygenic risk scores (PRSs) and rare high-risk monogenic variants have been modelled based on the UK biobank [8]. In prostate cancer, the population without pathogenic variants in *BRCA1/2, CHEK2, ATM* and *HOXB13E* accounted for 98.0% of individuals and 95.8% of cases. Those without pathogenic variants but with an intermediate (10–90%) PRS accounted for 78.5% of individuals and 73.9% of cancers; family history was estimated for 8.1% of men [8]. In a population with a pathogenic gene variant and 10–90% PRS the relative risk was 2 times higher than in a population without a pathogenic variant and 10–90% PRS, and family history was estimated for 11.4%. In the small population of both a pathogenic variant and > 90% PRS (0.2% of all individuals), about 1% of prostate cancers were found and 17.7% had a family history [8].

A similar calculation for breast cancer was conducted by using *BRCA1/2* as high-risk genes and *CHEK2, ATM* and *PALB2* for intermediary risk genes [8]. Monogenic component was larger in breast than in prostate cancer [8]. Another UK Biobank based study reported similar results on the same cancers with minor differences in the monogenic gene selection (*BRCA1* and *CHEK2* were not used for prostate cancer) and PRS was divided by population tertiles [8, 9]. Personalized risk prediction is further improved if family history is added to the genetic scores [36]. PRS has been used together with genetic and hormonal/environmental data in improving breast cancer screening and risk prediction models which may suggest clinical utility for the PRS [37, 38].

Recent evidence has been convincing, although counter-intuitive, that familial risk and PRS are largely independent; in the FinnGen study of common diseases the PRSs explained 10% of the effect of first-degree family history, and first-degree family history 3% of PRSs [39]. The inheritance patterns of monogenic and polygenic diseases differ. For a dominant disease gene, half of the offspring receive the risk allele. Polygenic inheritance is defined through a top set of independent risk alleles that are associated with the disease; these range usually between top 1% and 10% which may encompass the risk alleles from tens to hundreds of SNPs. Offspring inherit on average 50% of their alleles from each parent. Thus, none of the offspring receive exactly the same parental set of risk alleles. While 50% of siblings share a monogenic risk allele, only 35% of the siblings shared the top 10% PRS and only 15% shared the top 1% PRS (examples are from cardiometabolic diseases) [40]. A further consequence of polygenic inheritance is that the above mean values (35% and 15%) hide the amount of large variation of sharing between the sibling pairs [40]. While polygenic inheritance may reasonably capture risks between first-degree relatives, large family clusters in multiple generations would be less likely. Testis and thyroid cancers did not show any multiplex families which could be due to the polygenic origin of their familial risk but also due to the rareness of these cancers. In the above UK study on testicular cancer 15 cases (6.3%) were found in multiplex families out of a total of 236 familial cases [28]. The UK study was exceptional in showing that the PRS and familial risks were largely overlapping [28].

In conclusion, we summarized the current knowledge on the possibilities in disease gene identification on cancers with high familial risks, in which little of the constitutional background is known. The rareness of the esophageal, stomach, small intestinal, testis, thyroid and bone cancers calls for collaborative efforts recruiting either all cancers or possibly early onset cases, irrespective of family history in order to secure a reasonable sample size. Familial cases or cases with a second primary cancer of the same type could be used for confirmation purposes.

## Data Availability

No datasets were generated or analysed during the current study.
